# Autism Spectrum Disorder-Related Syndromes: Modeling with *Drosophila* and Rodents

**DOI:** 10.3390/ijms20174071

**Published:** 2019-08-21

**Authors:** Ibuki Ueoka, Hang Thi Nguyet Pham, Kinzo Matsumoto, Masamitsu Yamaguchi

**Affiliations:** 1 Department of Applied Biology, Kyoto Institute of Technology, Matsugasaki, Sakyo-ku, Kyoto 603-8585, Japan; 2 Department of Pharmacology and Biochemistry, National Institute of Medicinal Materials, Hanoi 110100, Vietnam; 3 Division of Medicinal Pharmacology, Institute of Natural Medicine, University of Toyama, Toyama 930-0194, Japan

**Keywords:** autism spectrum disorder, *Drosophila melanogaster*, rodent, learning assay, social space assay, circadian rhythm

## Abstract

Whole exome analyses have identified a number of genes associated with autism spectrum disorder (ASD) and ASD-related syndromes. These genes encode key regulators of synaptogenesis, synaptic plasticity, cytoskeleton dynamics, protein synthesis and degradation, chromatin remodeling, transcription, and lipid homeostasis. Furthermore, in silico studies suggest complex regulatory networks among these genes. *Drosophila* is a useful genetic model system for studies of ASD and ASD-related syndromes to clarify the in vivo roles of ASD-associated genes and the complex gene regulatory networks operating in the pathogenesis of ASD and ASD-related syndromes. In this review, we discuss what we have learned from studies with vertebrate models, mostly mouse models. We then highlight studies with *Drosophila* models. We also discuss future developments in the related field.

## 1. Introduction

Autism spectrum disorder (ASD) is characterized by impaired social interactions and communication as well as restricted, repetitive, and stereotyped patterns of behaviors, interests, and activities. Comorbidities occur in some ASD cases, including language deficits, epilepsy, intellectual disability, motor abnormalities, anxiety, and gastrointestinal issues. The estimated prevalence of ASD may be 1:59 children and 1:100 adults. ASD is suggested to be more common in males with a male:female ratio of 4:1.

Genome wide nucleotide sequencing analyses by deep sequencing identified many genes associated with ASD and ASD-related syndromes [1,2,3]. These genes have a wide variety of functions in synaptogenesis, synaptic plasticity, cytoskeleton dynamics, protein synthesis and degradation, chromatin remodeling, transcription, and lipid homeostasis [1,2,3]. Furthermore, in silico studies suggest complex regulatory networks among these genes. In the last decade, animal models have contributed to increased research on ASD and ASD-related syndromes. The *Drosophila* model is useful for clarifying not only the in vivo roles of ASD-associated genes, but also the complex gene regulatory networks among ASD-associated genes [3,4,5]. We herein review advances in our understanding of ASD-associated genes in both rodents and *Drosophila* models.

## 2. Syndromic Forms of ASD and ASD-Associated Genes

ASD has a complex genetic etiology, and monogenic disorders associated with the high penetrance of ASD are observed in less than 20% of ASD cases. Well-known single gene disorders associated with ASD are Fragile X syndrome, Rett syndrome, MECP2 duplication, Angelman syndrome, and Tuberous sclerosis [1].

Fragile X syndrome is caused by the expansion of a CGG triplet repeat in the 5′-untranslated region of the *Fragile X Mental Retardation gene 1* (*FMR1*) gene, leading to the hypermethylation of its promoter accompanied by transcriptional inhibition. The estimated prevalence of ASD is 30~60% (males only) [1]. The *FMR1* gene product exhibits RNA-binding activity and regulates the translation, stability, and transport of many of the mRNAs required for synaptic plasticity and other functions. More than 800 distinct mRNA targets of FMR1 have been identified to date and appear to be involved in various aspects of brain development and function [6].

Rett syndrome is a progressive neurological disorder caused by loss-of-function mutations in the *methyl-CpG-binding protein 2* (*MECP2*) gene. MECP2 functions as a global transcriptional regulator in both the suppression and activation of target genes and also as a regulator of RNA splicing. The estimated prevalence of autism is 61% (females only) [1]. Duplication of the Xq28 region containing the *MECP2* gene induces MECP2 duplication syndrome. The estimated autism prevalence is more than 90% (males only) for this syndrome [1].

Angelman syndrome is a monogenic disorder with an estimated ASD prevalence of 34% [1]. Many cases of Angelman syndrome are caused by loss-of-function mutations in the maternal allele of the imprinted *Ubiquitin-protein ligase E3A* (*UBE3A*) gene. UBEA3A functions as both an E3 ligase in the ubiquitin proteasome pathway and as a transcriptional coactivator. The *UBE3A* gene is expressed in neurons, but not in glia in the hippocampus and cerebellum of the brain [7]. The paternal normal allele of *UBE3A* is silenced by long non-coding RNA. The overexpression of UBE3A also increases the risk of autism known as Duplication 15q syndrome [8].

Tuberous sclerosis complex (TSC) is an autosomal dominant genetic disorder characterized by benign tumors in the brain and other organs, epilepsy, and cognitive impairment. TSC is caused by mutations in the *TSC1* or *TSC2* gene encoding hamartin and tuberin, respectively. Biochemical studies revealed that these proteins dimerize and form a complex that is involved in the negative regulation of the mammalian target of rapamycin (mTOR) protein complex. The ASD prevalence in TSC is estimated to range between 36% and 50% [1].

**Figure 1:**
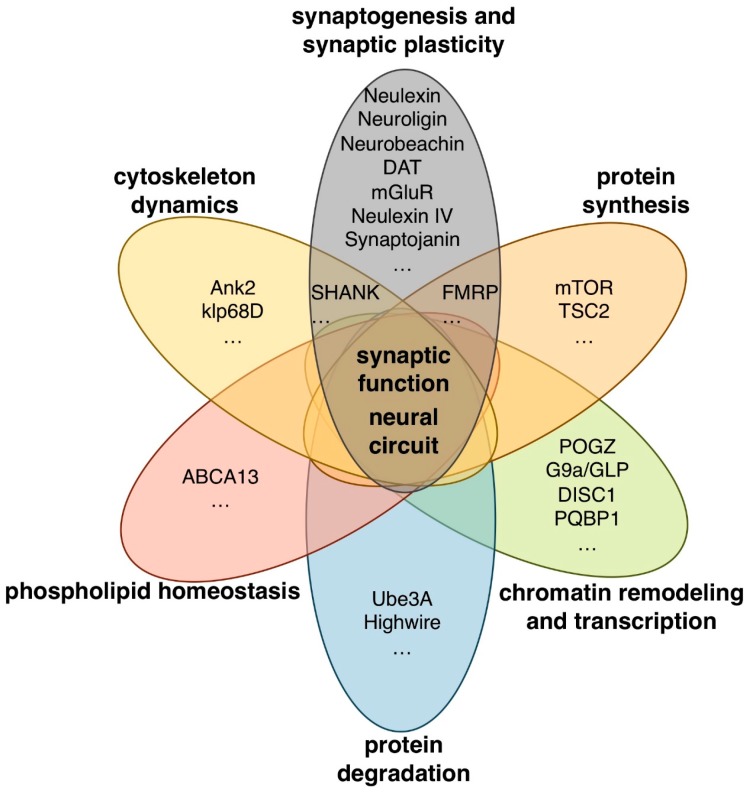
Human autism spectrum disorder (ASD)-associated genes with various biological functions.

Whole-genome and whole-exome sequencing analyses have identified many candidate ASD-associated genes to date [9,10,11,12,13,14]. These genes have a wide variety of functions including chromatin remodeling and transcription (*POGZ*, *G9a*, *GLP*, and *DISC1*), protein synthesis and degradation (*mTOR*, *TSC2*, *UBE3A*, and *PAM/MYCBP2*), scaffolding and cytoskeleton dynamics (*SHANK3*, *NBEA*, and *ANK3*), and synaptogenesis and synaptic plasticity (*CNTNAP2*, *Neuroligin-3,-4*, *DAT*, and *mGluR*) (Figure 1).

Mutations in the genes encoding proteins related to chromatin remodeling and transcription have been detected in ASD patients. Mutations in *POGZ* encoding the heterochromatin protein 1α (HP1α)-binding protein have been reported in cases of ASD [12], intellectual disability [15], and schizophrenia [16]. Missense variants for genes encoding the histone H3 lysine 9 (H3K9)-specific methyltransferases GLP (EHMT1) and G9a (EHMT2) have also been identified as ASD-associated genes [17,18]. A previous study reported that the *Disrupted-in-schizophrenia-1* (*DISC1*) gene is associated with ASD [19]. DISC1 interacts with transcription factor 4 (ATF4)/CREB2 in the nucleus to activate its target genes, which may be related to neuronal functions.

Mutations in the genes encoding proteins involved in protein synthesis and degradation are sometimes related to ASD. The mTOR pathway controls global mRNA translation and plays a role in various biological processes, such as autophagy, transcription, cytoskeletal dynamics, and neuronal differentiation [20]. mTOR interacts with TSC1/Hamartin and TSC2/Tuberin, as described above. Hyperactivation of the mTOR pathway increases the risk of ASD [21]. In addition to UBE3A, PAM/MYCBP2 (mouse Phr1 and *Drosophila* Highwire) is also a highly-conserved E3 ligase. TSC is also regulated by Phr1. A mutation in TSC2 related to tuberous sclerosis causes increased ubiquitination by Phr1 [22] that results in the enhanced degradation of TSC2 by Phr1.

The proper assembly of scaffolding proteins and the actin cytoskeleton are important for the proper positioning of cell-adhesion proteins, receptors, and channels at the synapse. Mutations in the genes required for these processes are also found in ASD patients. The SHANK family gene *SHANK3* is an ASD-causing gene [23,24]. Shank family proteins carry multiple domains including ankyrin repeats, SH3, PDZ, proline-rich, and SAM domains, and are the important organizers of postsynaptic density. Shank family proteins play a role in coordinating presynaptic and postsynaptic signaling through Neurexin–Neuroligin signaling complexes [25]. Disruption of the *Neurobeachin* (*NBEA*) gene by chromosomal translocation has been reported in patients with ASD [26]. *NBEA* encodes a signal scaffold protein carrying multiple domains that is mainly expressed in the brain during development [27]. *ANK3* encodes ankyrin-G, a scaffolding-adaptor that connects membrane proteins to the actin and β-spectrin cytoskeleton in order to allow proteins to assemble into discrete domains at the plasma membrane. Mutations in the *ANK3* gene have been identified in patients with ASD but are rare [28]. ANK3 regulates the structure and function of glutamatergic synapses [29].

ASD is also associated with various proteins involved in neuronal connectivity and synaptic transmission. The *contactin-associated protein-like 2* (*CNTNAP2*) gene, one of the neurexin family genes, appears to be one of the major susceptibility genes for various neurological disorders including ASD [30]. The cell adhesion glycoprotein CASPR2 encoded by *CNTNAP2* is involved in cortical neuron axon growth [31]. Neuroligins are type I membrane proteins consisting of a single large extracellular domain with a constitutively dimeric, enzymatically inactive esterase-homology domain and short cytoplasmic tail. There are four neuroligins in vertebrates, neuroligins-1, -2, -3 and -4, which are postsynaptic cell adhesion molecules that shape the functional properties of synapses [32]. Neuroligin-1 is localized in excitatory synapses and neuroligin-2 in inhibitory, dopaminergic, and cholinergic synapses [32]. Neuroligin-3 is expressed in excitatory and inhibitory synapses and neuroligin-4 in glycinergic synapses [32]. Mutations in neuroligins-3 and -4 have been associated with ASD [33,34]. Furthermore, mutations in genes encoding proteins involved in synaptic transmission have been associated with ASD, such as synaptic receptors, components in synaptic vesicle cycling, and ion channels. Abnormal signaling mediated through the *metabotropic glutamate receptor 5* (*mGluR5*) gene linked to the mTOR pathway is known to be involved in the pathophysiology of ASD [35]. Moreover, some genes that play roles in the dopamine network are associated with ASD, such as the plasma membrane protein syntaxin 1 (STX1) [36] and the dopamine transporter (DAT) [37].

A relationship has been reported between ASD and one of the A-subclass of ATP-binding cassette (ABCA) transporters, ABCA13. The mammalian ABCA protein family generally carries two transmembrane domains (TMDs), each of which includes six transmembrane domains and two cytoplasmic nucleotide-binding domains (NBDs) that contain the characteristic ATP-binding Walker A and B motifs. ABCA13 has the typical structure of ABCA family proteins, consisting of two TMDs and two NBDs, sharing strong similarities with some of the ABCA proteins, ABCA12 with 59%, ABCA1 with 51%, ABCA4 with 51%, and ABCA7 with 50% [38]. The transport substrates for ABCA12 are reportedly lipids, glucosylceramide, cholesterol, and phosphatidylcholine (PC) for ABCA1, N-retinylidene phosphatidyl ethanol amine for ABCA4, and PC for ABCA7. Based on amino acid sequence homologies, ABCA13 is also predicted to transport some lipid molecules [38]. In addition, ABCA13 is localized in neurons in the cerebral cortex, hippocampus, and cerebellum [38]. Genome-wide association studies with human patients implicated some rare coding variants of ABCA13 in the risk of neurological disorders, such as schizophrenia, bipolar disorder, and depression [39]. A recent study demonstrated that Japanese macaques exhibiting the ASD-like phenotype, such as impaired social ability as well as restricted and repetitive behaviors, carried rare coding variants in the *ABCA13* gene, which also suggested a link to ASD [40].

## 3. Vertebrate Models of ASD-Related Syndromes

Vertebrate models have been useful for understanding the etiology and pathogenesis of human diseases. Mice, rats, guinea pigs, voles (*Microtus*), and zebrafish (*Danio rerio*) have already been developed as vertebrate models of ASD by specific behavioral parameters. In this review, we mainly describe rodent models of ASD-related syndromes. To assess animal behavior in autism models, a set of tests is employed to assess behavioral changes in animals. The development of animal behavioral tasks relevant to the symptoms of autism is important to clarify ASD. An ideal mouse model of ASD with face validity needs to display behavioral abnormalities related to core symptoms, such as social and communicative impairments, restrictive repetitive behaviors, or different ways of learning and paying attention [41].

### 3.1. Assay Systems for Rodent ASD Models

We summarize several assays commonly used to characterize rodent ASD models.

#### 3.1.1. Sociability Test

Impaired social interactions are a hallmark feature of autism [42,43]. In experimental animals, they may be elucidated as alterations in social affiliation behavior using a number of experimental paradigms, i.e., the three-chamber test and resident–intruder test. 

The three-chambered test: This is an experimental paradigm that evaluates social approach behaviors in mice. The apparatus used in this test consists of three chambers separated by two opaque gates. Two identical transparent cylinder cages (or wire-cup cages) are placed on each side chamber. The test consists of training and test trials. In the training trial, subject animals are individually placed in the center chamber of the apparatus for 5 min to acclimatize them to the experimental arena and procedures. In the test trial, a stranger naïve mouse is placed into one of the cages as a social stimulus, while the other cylinder remained empty. The behavior of the subject mouse is video-recorded during a 10-min observation period. Social behavior of a subject mouse is measured by comparing the times the animals spent exploring around each chamber. [44,45,46,47]. 

The resident–intruder test: A subject mouse is placed in a neutral cage as a resident before the test [45,46,48]. After a period of acclimatization, an age- and sex-matched naïve mouse is placed in the cage as an intruder. The total cumulative duration of time the subject mouse spends sniffing the intruder mouse is measured as an index of social behavior.

The open field test: Social interactions between the subject and naïve animals in a square arena may be used as an index of the social performance of experimental animals [46,49]. In this test, the subject animal is placed in the arena for 5 min, for example, for habituation, and a naïve control animal is then placed in the arena. The total cumulative duration of the time the subject animal spent sniffing the naïve control is recorded as an index of social interaction behavior.

#### 3.1.2. Open Field Behavior Test

This test is employed to elucidate the anxiety-related behavior and stereotyped grooming behavior of rodents [46,49]. Animals are placed individually in an open field box (50 × 50 × 50 cm) with 1 lux lightness for 15 min. The first 10-min observation period may be used to measure the total duration of time each animal spent in the center zone as an index of anxiety-related behavior. The latter 10-min period between 5 and 15 min after starting the test is used to measure stereotyped grooming behavior as an index of restrictive repeated behavior related to a core symptom of ASD.

#### 3.1.3. Learning and Memory Tests

Different types of tests are used to elucidate the learning and memory performance of rodent models including ASD. The following are examples.

Modified Y maze: This test is used to evaluate the short-term spatial working memories of mice [46]. The apparatus consists of black polypropylene walls with 3 arms. This test consists of sample and test trials that are separated by an inter-trial interval. In the sample test, each mouse is individually placed in the Y-shaped maze with one of the 3 arms closed. The animal is allowed to explore the other 2 arms freely for 5 min. In the test trial that is conducted 30 min after the sample trial, the animal is again placed in the maze with all 3 arms opened, and allowed to explore the arms freely. The time animals spent in the novel arm that had been closed in the sample trials is recorded as an index of spatial working memory.

Fear-conditioning test: This test is an associative learning task in which subject animals acquire memory in association with a conditional stimulus, such as environment and tone. This test is used to elucidate long-term fear memory performance. The chamber for fear conditioning consists of a clear acrylic chamber and a stainless-steel grid floor equipped with an electric shock generator/scrambler. In the training trial, animals are placed individually and receive an acoustic tone that co-terminates with electric foot shocks. The tone-foot shock pairing is repeated five times at 1-min intervals. Contextual and auditory fear memories are elucidated 1–5 days after the training trial. To analyze the contextual memory, mice are placed in the same chamber and allowed to move freely for 6 min. One minute after placing the animal in the chamber, freezing behavior during a 5-min period is recorded as an index of contextual fear memory. To analyze auditory-dependent fear memory, mice are placed in the chamber for 6 min. After a 3-min habituation period, the tone is delivered continuously for 3 min. Freezing behavior during the 3-min period is recorded as auditory-dependent fear memory. Freezing is defined as the absence of any movement, except for that related to respiration [44,48].

Water finding test: This test is employed to analyze latent learning behavior, which is related to attention deficit-like behavior [48,50]. The apparatus used for this test consists of an open field compartment, a hollow on the compartment wall, and a drinking nozzle for tap water set on the ceiling of the hollow. The drinking nozzle is set 5 and 7 cm above the floor in the training and test trials, respectively. In the training trial, each mouse is placed in one corner of the open field and allowed to explore freely for several minutes. Subject mice are then deprived of water for 24 h. In the test trials, the animals are again placed individually into the apparatus and the latencies to enter the hollow and drink water (drinking latency) are measured as an index of latent learning behavior.

### 3.2. Rodent Models of ASD and ASD-Related Syndromes

There are generally two types of rodent models for ASD, non-genetic and genetic models [51,52,53]. In this review, we focus on several rodent ASD caused by the factors described above.

#### 3.2.1. Non-Genetic Rodent Models of ASD

Because of the high heritability of ASD, lines of studies have been focusing on genetic animal models which possess genetic alterations related to ASD. However, increasing prevalence or heritability of ASD have highlighted the necessity of other risk factors such as environmental factors (parental age, fetal environment, sex steroids, infections, medication, etc.), epigenetic factors, or interaction between environmental and genetic factors as well [34,54].

Non-genetic animal models of ASD are induced by prenatal exposure to chemical agents such as valproic acid (VPA), thalidomide, and ethanol during pregnancy, viral infections, or inflammation [53,55,56,57]. Among these factors, VPA exposure during pregnancy significantly increases the rate of ASD in offspring [55,58]. VPA is an anti-epileptic and mood-stabilizing drug administered to patients during pregnancy. Clinical research has identified the risks associated with the use of VPA, including birth defects, congenital malformations, developmental delays, reduced cognitive function, and autism [59,60]. VPA-induced autism is likely caused by defects in neuronal development of the cerebellum, limbic system, and brain stem to induce a disruption in synaptic connectivity.

In the animal model, the exposure of rodents to VPA may induce behavioral abnormalities as well as changes in neuroanatomy at the cellular level and gene expression that are similar to those observed in ASD patients [61,62]. The time and dosage of VPA as well as the route of exposure vary widely among studies [52]. The majority of studies examined maternal rodents at approximately day 12.5 of exposure to VPA at doses of 500–600 mg/kg (i.p). At this point, this dose causes similar abnormalities to autism in animals. Additionally, sex differences need to be considered in the VPA model. Clinically, the prevalence of ASD in children was found to be 3.46-fold higher in boys than in girls [63]. The majority of studies on VPA-induced ASD in rodents have also shown that VPA-treated male animals show greater impairments in social interactions, while other behaviors, such as repetitive/stereotyped, anxiety-like behaviors, and locomotor activity may or may not differ [64,65,66]. These findings suggest that social interactions are the most sensitive behavioral domain affected in a sex-specific manner [67].

VPA-induced ASD-like rodent models have been used to test various pharmacological reagents in order to develop new ASD therapy [68]. These pharmacological studies have mainly targeted classical neurotransmitter systems, endocannabinoids, the Wnt signaling pathway, and neuroinflammation [68].

SKF105111 (SKF), a selective type I 5α-reductase inhibitor, may induce ASD-like behaviors in male mice, such as impaired sociability-related performance and repetitive grooming behaviors [45]. SKF was shown to decrease the endogenous levels of allopregnanolone, a positive allosteric modulator of the GABA_A_ receptor, and, thus, reduce GABAergic synaptic transmission in the brain [69]. A rationale of this model is based on pathophysiological studies on ASD, indicating that dysfunctions in the GABAergic system in the brain are one of the major causes of ASD symptoms [70,71]. In a study using SKF-treated male mice as an animal model of ASD, Kamishoyosan, a traditional Kampo formula used for the treatment of menstrual irregularity-related symptoms and menopause-related symptoms, was found to ameliorate ASD-like behaviors by facilitating dopamine receptor-mediated mechanisms and partly by neurosteroid-independent GABA_A_ receptor-mediated neurotransmission [72]. 

#### 3.2.2. Genetic Rodent Models of ASD and ASD-Related Syndromes

The ASD genetic models induced in mice may be established by targeting a mouse homologue of human ASD-causing genes. The established monogenic mouse models in many cases reflect key aspects of human ASD symptoms and respond to pharmacotherapies that may be operational in human ASD. Since a number of mouse ASD models targeting various ASD-causing genes have been developed and extensively reviewed on their usefulness and limitations [53,73,74,75], we herein briefly summarize several examples that are also modeled in *Drosophila* as described below.

*FMR1* knockout male mice show social interaction deficits, hyperactivity, and cognitive impairments [75]. In this model, *FMR1* was required for the development of mature and stable dendritic spines in the brain [53]. Dysfunctions in mGluR5 were also observed in this model [75,76]. Furthermore, social interaction deficits in *FMR1* knockout mice may be due to the down-regulated expression of neuroligin 1 because these deficits were rescued by the overexpression of neuroligin 1 [77]. The limitation of *FMR1* knockout mice is that their ASD-like phenotypes extensively vary depending on their genetic background [78]. The *FMR1* knockout mice on the B6D2 background are suggested to be an ideal model in studying a role of *FMR1* in ASD, since they show behavioral defects consistent with core symptoms of ASD [78].

*MECP2* conditional mutant mice show repetitive, stereotypic, and restricted behaviors, an abnormal gait, reduced anxiety, decreased pain, and normal olfactory discrimination [79,80]. *MECP2* mutant mice show decreased spine density in cortical neurons and decreased dendritic complexity [81]. Among the genes that are dysregulated up and down in *MECP2* mutant mice, the gene encoding the neurotrophic factor BDNF, which is down-regulated in this model mouse, may be one of the critical MECP2 target genes because treatments that increased BDNF in mice attenuated their symptoms [82]. Various *MECP2* conditional mutant mice revealed that the loss of MECP2 in a subset of excitatory or inhibitory neurons induced ASD-like features [75,79].

Angelman syndrome is a monogenic disorder with an ASD prevalence of approximately 34% [1]. Most cases of Angelman syndrome are caused by loss-of-function mutations in the maternal allele of the imprinted *UBE3A* gene, the gene product of which carries a HECT domain driving ubiquitylation to mediate substrate degradation by the proteasome. In contrast, Dup15q is an autism-associated disorder co-incident with high rates of pediatric epilepsy. Additional copies of the *UBE3A* gene are considered to induce Dup15q phenotypes [8]. *UBE3A* knockout mice show impaired social interactions, repetitive behavior, and restricted interest [75,83]. The *UBE3A* knockout mice have decreased spine density in cortical neurons [7]. It has also been demonstrated that UBE3A is required for apical dendrite outgrowth [84]. Many target proteins of UBE3A-dependent ubiquitination have been identified to date. The activity-regulated cytoskeleton protein (Arc), which is responsible for the internalization of AMPA receptors, is one of the target proteins [85]. This glutamate receptor mediates synaptic transmission in the CNS. It is also important to note that *UBE3A* knockout mice show defects in BDNF signaling [86].

Although the homozygous deletion of *TSC1* or *TSC2* results in embryonic lethality, heterozygous mice recapitulate many phenotypes of human TSC patients, including ASD-like behaviors such as social interaction deficits and repetitive and restricted behavior or interest [87]. In this mouse model, soma size is increased, whereas changes in spine density vary developmentally and across brain regions [75]. The hyperactivation of mTORC1 is also observed in this mouse model [88]. It is important to note that long-term potentiation initiated by BDNF is also dependent on mTOR signaling [75].

The *NBEA* gene has been identified as an ASD candidate gene in four unrelated patients that show haploinsufficiency for the *NBEA* gene. The haploinsufficiency of *NBEA* may induce cognitive dysfunction and ASD-like phenotypes, including changes in self-grooming behavior and deficits in social behaviors, conditioned fear responses, and spatial learning and memory in mice [89]. The alterations in learning and memory observed in this mouse model are accompanied by decreases in the expression levels of the immediate early gene *zif268* in the dorsomedial striatum and hippocampal cornu ammonis 1 region, the increased phosphorylation of cAMP response element-binding protein (CREB), and increases in hippocampal BDNF expression [89]. Thus, *NBEA* haploinsufficiency affects neuroplasticity and behavioral functions in mice, underlying ASD symptoms in NBEA-deficient humans. The loss of *NBEA* has been shown to cause the abnormal clustering of synaptic proteins on dendritic shafts and decreases in actin in spines [90]. Therefore, *NBEA* deficiency may induce spine loss and corresponding defects in synaptic efficacy and plasticity [91]. Previous studies with *NBEA* knockout mice also revealed a role for NBEA in neurotransmitter release and synaptic function [92]. 

## 4. *Drosophila* Models of ASD and ASD-Related Syndromes

*Drosophila* homologues of some human ASD-associated genes that have a wide variety of functions in synaptogenesis, synaptic plasticity, cytoskeleton dynamics, protein synthesis and degradation, chromatin remodeling, transcription, and lipid homeostasis are summarized in Figure 1. *Drosophila* models of ASD and ASD-related syndromes targeting some of these genes and their assay systems are described below. 

### 4.1. Assay Systems for Drosophila ASD Models

We summarize several assays commonly used to characterize *Drosophila* ASD models. This may be useful for beginners who want to start using *Drosophila* as a model in studies on human ASD.

#### 4.1.1. Social Space Assay

**Figure 2:**
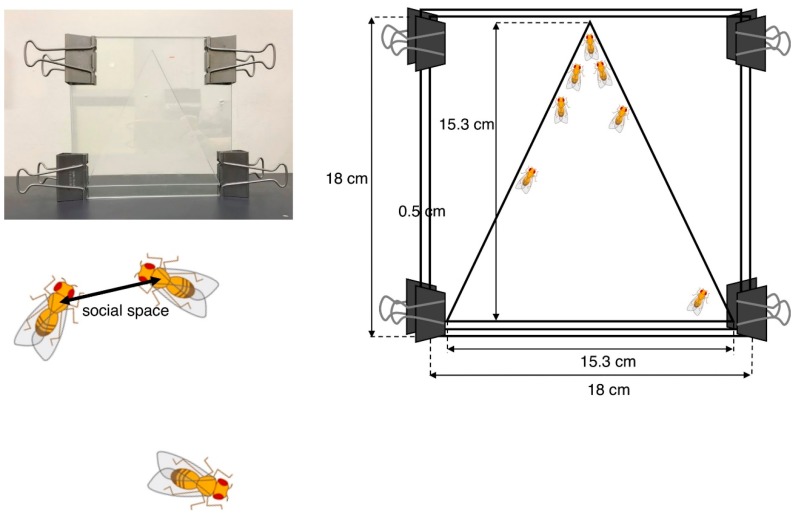
Social space assay. The vertical triangle test chamber is shown in the photo (**left**) and drawing showing its dimensions (**right**). The social space indicates the distance to the nearest neighbor.

Social space assays were originally developed by Simon et al. to evaluate the social activity of adult ASD model flies [93] and are now commonly used in the characterization of various *Drosophila* ASD models. The test chamber contains two square glass plates (18 × 18 cm) separated by a 0.5-cm spacer allowing flies to be in a space. The internal space has 15.3 cm of height and base (Figure 2). 

Newly eclosed adult flies were placed in food vials at 25 °C and raised for 3 to 4 days. Male and female flies were then separately placed in other food vials (40/vial) 1 day prior to the experiment. These flies were placed in the chamber at midday siesta (3–4 h after the start of the light cycle) to minimize possible variations caused by the circadian rhythm. The bottom of the chamber was then banged to set all flies climbing up from the same starting point in the bottom. After 20 min, all flies settled at some positions in the chamber. The nearest neighbor distances were analyzed by ImageJ software after importing the images by digital camera. In order to eliminate outliers, the distribution of distances was represented using boxes and Tukey’s whisker plots.

#### 4.1.2. *Drosophila* Activity Assay

The *Drosophila* activity assay is commonly used to evaluate the activity and circadian sleep–wake rhythm of ASD model flies [94,95]. Newly eclosed adult male flies were used in this assay. The 3-day-old male flies were placed in *Drosophila* Activity Monitors (Trikinetics) that are installed in the 25 °C incubator. Flies were then monitored by this equipment for 6 consecutive days under a 12-h light–dark cycle. The fly activity recorded for each 15- or 30-min bin was evaluated by how many times per 15 or 30 min each individual fly crossed an infrared light beam irradiated at the center of the capillary tube. The values were shown in the graph at the endpoint of the 15- or 30-min measurement as the average activity bouts of flies.

#### 4.1.3. Odor–Taste Learning Assay in Larvae

**Figure 3:**
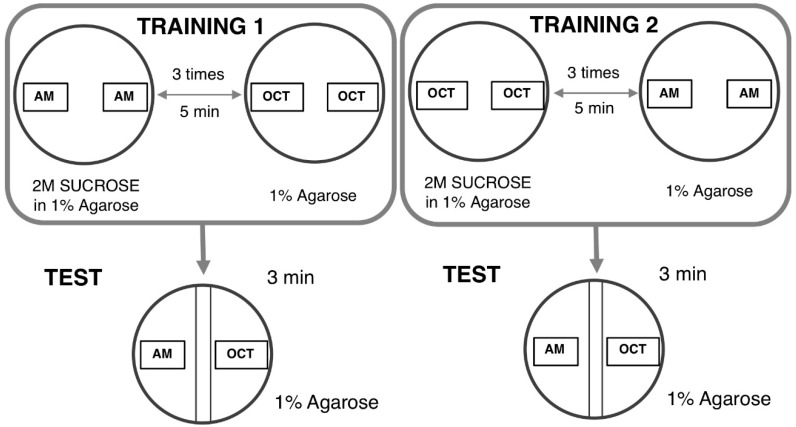
Principle of the odor-taste learning test. TRAINING 1: In one of the groups, n-amyl acetate (AM) is added with a sucrose reward and 1-octanol (OCT) is subsequently added without a reward. TRAINING 2: The other group receives reciprocal training. TEST: After each training exposure, larvae are tested for their choice between AM and OCT.

The larval learning assay based on odor–taste is commonly used to evaluate the learning ability of *Drosophila* ASD models [96,97]. The learning assay is based on two distinct steps as follows. In the first step, a group of larvae was exposed to n-amyl acetate (AM) (Millipore, 818700) in the presence of a reward (2 M sucrose, SUC) and then followed by 1-octanol (OCT) (Sigma) in the absence of SUC. This set of training was defined as AM+/OCT, indicating “+” as the reward (Figure 3). 

In the next step, reciprocal training was performed. A group of larvae was sequentially exposed to OCT in the presence of SUC followed by AM exposure in the absence of SUC. This second set of training was defined as OCT+/AM. Five minutes of each training allowed flies to establish an association between AM or OCT and the reward (SUC). These sets of training were repeated three times. Ten microliters of each odorant were added in 0.2-mL Eppendorf tubes with a perforated lid and placed on the opposite site inside a proper Petri dish filled with 1% agarose. Normally undiluted AM and OCT diluted at a 1:75 with liquid paraffin were used for this test. Two agar plates were used for the training, one for each of two odorants. Each agar plate was divided into two distinct zones with a 1-cm neutral zone in the middle. The neutral zone was defined as the area where larvae received the same intensity of each odorant. Twenty-four larvae for each corresponding training (AM +/OCT and OCT+/AM, respectively) were divided into three groups with 8 larvae. Each group then received training in the appropriate agar plate. After training, larvae in groups of 8 were transferred into a plate without SUC in which the odorants were placed on the opposite ends. The preference indexes were used to calculate the learning index (LI). The AM preference was calculated as follows: The Number of Larvae on the AM side – the Number of Larvae on the OCT side that was divided by total number of larvae on both sides. The AM preference can range from 1 (indicating perfect attraction to AM) to −1 (indicating perfect attraction to OCT). Normalized AM LI was calculated as follows: AM preference – Average of OCT preference that was divided by two. Normalized OCT LI was calculated as follows: Average AM preference – OCT preference that was divided by two. LI was calculated as Normalized AM + Normalized OCT that was divided by two.

#### 4.1.4. Visualization of NMJs by Super Resolution Microscopy

**Figure 4:**
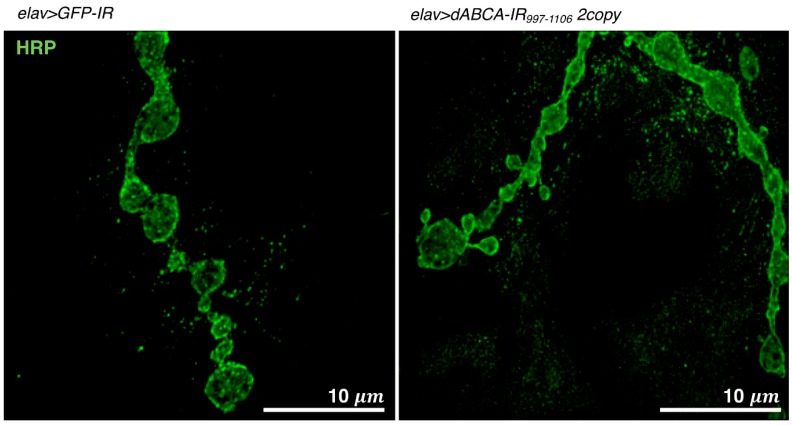
Visualization of NMJs in muscle 4 of a third instar larva. Images show NMJs that were stained with anti-HRP IgG. Images were taken using a super-resolution microscope (N-SIM, Nikon). *elav>GFP-IR*, *w/Y; UAS-GFP-IR/+; elav-Gal4/+. elav>dABCA-IR_997-1106_ 2copy*, *w/Y; UAS-dABCA-IR_997-1106_/UAS-dABCA-IR_997-1106_; elav-Gal4/+.* Bars indicate 10 μm.

The neuromuscular junction (NMJ) in *Drosophila* ASD models is commonly visualized using laser confocal microscopy; however, its visualization by super resolution microscopy (N-SIM, Nikon) has recently been performed, as described in [95,98]. In structured illumination microscopy (SIM), the cellular ultrastructure is examined by analyzing the moire pattern produced when illuminating a specimen with a known high-frequency patterned illumination. N-SIM shows super resolution of up to 115 nm in multiple colors. Third instar larvae were washed with *Drosophila* ringer and dissected in HL3 saline. Dissected larvae were fixed on a 5.0-cm plastic petri dish by small pins. The movie for the dissection of NMJ is available at this site (https://www.ncbi.nlm.nih.gov/pmc/articles/PMC2762896/) [99]. Dissected larvae were fixed in 4% paraformaldehyde in PBS and then stained with FITC-conjugated anti-HRP IgG. After washing, samples were mounted with ProLong Diamond (Invitrogen) for N-SIM. An example of a visualized NMJ is shown in Figure 4.

#### 4.1.5. Electrophysiology at the NMJ

The *Drosophila* NMJ has commonly been used as a model to study the molecular mechanisms underlying synaptic transmission. Electrophysiological analyses at the NMJ provide information on neurotransmitter release from presynaptic nerve terminals to activate glutamate receptors at the motor endplate on the muscle and are useful for characterizing *Drosophila* ASD models. In *Drosophila*, the postsynaptic membrane is designated as the subsynaptic reticulum. Glutamate receptors localizes opposite the active zones. Glutamate binds to the glutamate receptor that allows Ca^2+^ to enter the muscle in order to depolarize the muscle membrane. *Drosophila* provides a suitable model for the study of the NMJ because an action potential is absent in *Drosophila* muscle. In the case of mice, the action potential has to be prevented by blocking Na^+^ channels in order to measure the endplate potential. *Drosophila* third instar larval NMJs may be measured at 25 °C, which maintains their electrical properties for several hours. The preparation of a recording microelectrode, stimulation microelectrode, or suction electrode, dissection of the 3rd instar larva body wall, electrophysiology recordings, and analyses of neurotransmitter release and quantal contents were previously reported [100].

### 4.2. Drosophila Models Targeting FMR1

As described above, the estimated ASD prevalence in Fragile X syndrome is 30~60% (males only) [1]. Humans carry three FMR1 family proteins: FMR1, FXR1, and FXR2. *Drosophila* contains a single FMR1 homologue, dFMR1, sharing a similar level of sequence homology with all three human paralogues, but is functionally the most closely related to human FMR1 [101]. dFMR1 is mainly expressed in the CNS. The *dFMR1* mutants initially generated were viable and fertile, similar to humans [102]. However, the viability of the mutant appears to be sensitive to the genetic background because some *dFMR1* mutants may become lethal under some genetic backgrounds in a generation-dependent manner [103]. Loss-of-function mutants of *dFMR1* exhibit abnormal synapse structures with overgrowth, over-branching, and increased synaptic boutons in peripheral NMJs as well as in the mushroom bodies (MB) of the CNS, which are also accompanied by altered neurotransmission [102,104]. Among the three human paralogues *FMR1*, *FXR1*, and *FXR2*, only *FMR1* was able to rescue the abnormal synapse structure in *dFMR1* null mutants [105]. The pre-synaptic requirement of dFMR1 for synapse morphogenesis, along with pre- and post-synaptic requirements for functional neurotransmission have also been reported [106]. Furthermore, loss-of-function mutants of *dFMR1* exhibit increased dendritic branching in dendritic arborization neurons and its role in dendrite development appears to be partially mediated by Rac1 as well as microRNA [107,108]. In addition, defects in axonal targeting have been reported in *dFMR1* mutants [103,104,109,110,111]. 

RNA immunoprecipitation identified *futsch* as a target of dFMR1 [102]. *futsch* is a *Drosophila* homologue of mammalian MAP1B encoding a microtubule-associated protein. A mutation in *futsch* results in the undergrowth of synaptic boutons, while the *dFMR1* mutation induces the overgrowth of synaptic boutons. In addition, dFMR1 binds futsch mRNA to inhibit its translation [102]. dFMR1 may also bind to the mRNAs of BMPR2 [112] and DSCAM [113] as well as to the Ca^2+^/calmodulin dependent protein kinase II mRNA together with Ataxin-2 [114], suggesting the role of dFMR1 in the Ca^2+^ signaling pathway. The larval NMJ is a useful system for examining genetic interactions in vivo. In *Drosophila* NMJs, *dAdar* acts downstream of *dFMR1* to generate a proper NMJ structure [115]. Human Cytoplasmic FMR1 Interacting Protein 1 (CYFIP1) is reported to be involved in neurodevelopmental disorders, such as intellectual disorder, autism, schizophrenia, epilepsy, and Burnside–Butler syndrome [116,117,118,119]. In *Drosophila*, dFMR1 and dCYFIP1, a *Drosophila* homologue of CYFP1, play opposing roles in larval NMJ length. dFMR1 represses, while dCYFIP1 promotes synaptic growth at the NMJ [120]. In double homozygous mutant flies, synapse morphology phenotypes at the NMJ were mutually rescued. Many other target genes of dFMR1 have also been identified, such as genes encoding the synaptic heparan sulfate proteoglycans glycosylphosphatidyl inositol-anchored Dally-like protein and the transmembrane Syndecan, which play critical roles in modulating the synaptic structure and functions [121,122,123]. Previous studies reported that the expression of these two genes increased in the NMJ of *dFMR1* mutants [112,113].

Several pharmacological approaches have been taken with model flies targeted to *dFMR1* [121]. *dFMR1* mutants show less vigorous courtship behavior and defects in short-term memory and circadian rhythm [109]. The treatment of larvae and adults with the non-competitive mGluR antagonist 2-methyl-6-(phenylethynyl)pyridine, three competitive mGluR antagonists, or LiCl rescued defects in the courtship behavior and memory of *dFMR1* mutants [124]. The treatment of model flies with an inhibitor of LIMK1, a downstream target of BMPR2, restored larval bouton numbers and the locomotive defect in *dFMR1* mutants that was evaluated by the larval crawling assay [125]. In addition, the treatment of dFMR1 mutants with metformin restored short-term courtship memory and long-term olfactory memory [126]. Metformin is generally administered for type 2 diabetes and acts as a sensitizer of insulin signaling by increasing PTEN expression, activating AMPK, and decreasing TOR signaling. Thus, model flies targeted to *dFMR1* may be useful for the screening of candidate drugs for Fragile X syndrome and ASD.

### 4.3. Drosophila Models Targeting UBE3A

*Drosophila* UBE3A (dUBE3A) contains a C-terminal HECT domain consisting of 350 amino acids that shares 62% amino acid identity with human UBE3A. It is ubiquitously expressed during early embryogenesis, including the developing nervous system [127]. In the adult brain, dUBE3A is widely expressed, including in MB, a well-known center for learning and memory [128]. *dUBE3A* null mutants show no defect in viability, but have behavioral defects, including deficiencies in climbing ability, the circadian rhythm, and long-term associative olfactory memory [128]. The loss of dUBE3A activity induced a reduction in the dendritic branching of sensory neurons in the peripheral nervous system and slowed the growth of terminal dendrites. Moreover, the overexpression of dUBE3A decreased dendritic branching, indicating that the proper level of dUBE3A may be crucial for normal dendritic patterning [129]. Consistent with these findings, other studies revealed that the up- and down-regulation of dUBE3A were equally detrimental to learning in larvae and adults [130]. Studies with conditional gene expression revealed that dUBE3A was required for normal brain development and during adulthood [130]. The *dUBE3A* gene genetically interacts with other genes involved in learning and memory, such as the *derailed* gene [127]. Recent studies revealed that the glia-specific overexpression of dUBE3A by repo-GAL4 increased the levels of the sodium/potassium (Na^+^/K^+^) exchanger called ATPα, and this was accompanied by a robust seizure-like phenotype [131]. The glia-specific knockdown of ATPα also induced seizure-like behavior, and this phenotype was suppressed by the simultaneous overexpression of ATPα and dUBE3A in glia. Furthermore, the overexpression of *dUBE3A* in glia, but not neurons, impaired photoreceptor neuron function, thereby emphasizing the critical effect of *dUBE3A* overexpression [131]. Previous studies indicated that the overexpression of *dUBE3A*, but not the ubiquitination defective dUBE3A-C/A mutant form, compromised the ability of motor neuron axons to support closely spaced trains of action potentials, while simultaneously increasing excitability [132]. These studies using *Drosophila* models may contribute to our understanding of the defects in synaptic plasticity commonly observed in ASD.

Proteomic studies on the *Drosophila* brain overexpressing *dUBE3A* or human *UBE3A* identified proteins differentially driven toward polyubiquitination followed by degradation, such as Rho-GEF Pebble [127]. Tetrahydrobiopterin (THB) was identified in similar screening of the UBE3A-mediated modulation of the GTP cyclohydrolase Punch, an enzyme that produces the rate-limiting co-factor in monoamine biosynthesis [133]. The level of Punch was increased by the overexpression of *dUBE3A* and decreased by the mutation of *dUBE3A*. THB, neopterin, and dopamine levels in the brain were simultaneously increased by the overexpression of *dUBE3A* and decreased by the knockdown or loss-of-function mutation of *dUBE3A* [133]. These findings suggest that altered dopaminergic function contributes to the symptoms associated with UBE3A copy number variants (CNVs) that are related to ASD [134,135]. More recently, three proteasome-related proteins, Rpn10, Uch-L5, and CG8209, in addition to the ribosomal protein Rps10b were found to be directly ubiquitinated by dUBE3A in neuronal cells [136]. Among these, only Rpn10 was degraded upon ubiquitination by dUBE3A, suggesting that degradation is not the only effect of dUBE3A on its substrates [136]. Consistent with this finding, the genetic interaction between dUBE3A and the C-terminal part of Rpn10 was observed in vivo. The overexpression of these proteins enhanced the accumulation of ubiquitinated proteins, providing further biochemical evidence to support the interaction obtained in neuronal cells [136]. These biochemical and genetic studies with *Drosophila* models provide a basis for understanding the role of dUBE3A in ASD and Angelman syndrome.

### 4.4. Drosophila Models Targeting Neurobeachin (rugose)

The product of the ASD candidate the *Neurobeachin* (*NBEA*) gene plays multiple roles in the regulation of secretion, receptor trafficking, synaptic architecture, and protein kinase A (PKA)-mediated phosphorylation, as described above [26]. NBEA is a neuron-specific signal scaffold protein consisting of several distinct domains such as a concanavalin A-like lectin domain flanked by armadillo repeats (ACA), an A-kinase anchoring protein domain that has affinity to PKA, a domain of unknown function (DUF1088), and a BEACH domain, preceded by a pleckstrin homology-like domain with WD40 repeats (PBW) [137]. 

*Drosophila* rugose (rg) is a homologue of human NBEA, sharing high amino acid sequence conservation within the BEACH, pleckstrin homology-like, WD40 repeats, armadillo repeats, and concanavalin A-like lectin domains [138]. A previous study reported that rg is distributed in a granular pattern similar to the Golgi network in neuronal cell bodies [138]. Similar to many other ASD model flies, *rg* null mutants are viable and fertile, but exhibit defects in learning ability and changes in the gross brain morphology and synapse structure at the NMJ [138]. Other studies revealed that loss-of-function mutants of *rg* exhibited an abnormal synaptic architecture and physiology in larvae and defects in social interactions, impaired habituation, aberrant locomotion, and hyperactivity in adults [139]. These findings indicate that *Drosophila rg* mutants exhibit phenotypic characteristics similar to human ASD and, thus, may be a suitable model for studying ASD. Adult *rg* null mutants exhibit a small, rough eye phenotype accompanied by a disorganized retina and aberrant cone cell differentiation, resulting in the loss of cone cells [140]. *rg* genetically interacts with the genes encoding the components of the EGFR- and Notch-mediated signaling pathways, suggesting that *rg* is required for correct retinal pattern formation and may function in cell fate determination mediated by the EGFR and Notch pathways [140]. Later studies revealed that the loss-of-cone cell phenotype was due to cell type-specific apoptosis rather than transformation because the defect was rescued by a reduction in proapoptotic signals [141]. Moreover, the defect was rescued by an increased Notch signal, suggesting the importance of the anti-apoptotic function of Notch [141]. In addition, the loss-of-cone cell phenotype of *rg* mutants was shown to be accompanied by enhanced Jun N-terminal kinase activity and a simultaneous reduction in EGFR signaling activity [141]. A clearer understanding of the role of NBEA in ASD is needed, and further studies on the role of these signaling pathways in relation to the neural function of *rg* are warranted.

### 4.5. Drosophila Models Targeting ABCA

ABCA13, a member of the ABCA family of proteins, is predicted to transport lipid molecules. Whole-exome sequencing analyses of children with ASD identified *ABCA13* as one of the ASD-associated genes [11,12,14]. Furthermore, recent studies revealed that a monkey carrying the heterozygous *ABCA13* deletion and a mutation of the serotonin 2C receptor (*5HT2c*) displayed an impaired social interaction ability, an obsession with systems, and repetitive behavior, which are associated with ASD [40]. This monkey model of ASD with the *ABCA13* deletion and the mutation of *5HT2c* exhibited defects in neural maturation in the CNS [142]. *Drosophila* has a homologue for human ABCA family genes called *dABCA* (*CG1718*), which shows the highest homology to human *ABCA13*.

The asymmetric distribution of phospholipids across the plasma membrane and its local changes are important for cell functions. Based on amino acid sequence homologies to other ABCA family proteins that transport various lipid molecules, ABCA13 may also transport lipid molecules [38]. Since the CNS represents one of the most lipid-rich areas in higher organisms, ABCA transporters may play crucial roles in the integrity of the CNS. Recent studies indicate that ABCA transporters are involved in the maintenance of brain lipid homeostasis and neurodegenerative diseases, as suggested for other ABC protein family members [143]. Although transport substrates for dABCA currently remain unclear, dABCA may also transport lipid molecules, as noted for human ABCA family proteins, and may be involved in the maintenance of lipid homeostasis in the *Drosophila* brain.

A *Drosophila* ASD model targeting *dABCA* was recently established [95]. The pan-neuron-specific knockdown of *dABCA* by the elav-Gal4 driver increased social space with the closest neighbor in adult male flies, as evaluated by the social space assay, as described above [95]. An activity assay (Trikinetics) with adult male flies revealed that the pan-neuron-specific knockdown of *dABCA* induced hyperactivity all day accompanied by the early onset of evening anticipation in adult flies [95]. These phenotypes suggest that the established *dABCA* knockdown fly is a relevant model for ASD. An increase in satellite bouton numbers in the presynaptic terminals of glutamatergic neurons was also observed in *dABCA* knockdown larvae (Figure 4), suggesting that *dABCA* plays a role in the formation and/or maintenance of presynaptic terminals [95]. Genome-wide genetic screens using the established *dABCA* knockdown flies are now being undertaken to identify genes that genetically interact with *dABCA*. Once *dABCA*-interacting gene(s) are identified in *Drosophila*, their human homologue(s) may be candidate(s) for novel diagnostic marker(s) and/or therapeutic targets for ASD. As is the case with other ASD model flies, *dABCA* knockdown flies may also be suitable for the screening of candidate substances for ASD therapy.

### 4.6. Drosophila ASD and ASD-Related Models Targeting Other Genes

Defects in the function of dopamine have been implicated in various neuropsychiatric disorders, such as bipolar disorder, schizophrenia, ADHD, and ASD [144,145,146,147,148]. The whole-exome sequencing of ASD families identified a missense mutation in the *hDAT* gene encoding DAT [11]. DAT is a presynaptic membrane protein that regulates dopamine homeostasis in the CNS by mediating the re-uptake of released dopamine in synapses. A *Drosophila* model expressing *hDAT* carrying the T356M mutation has been established [94]. The dopaminergic neuron-specific expression of *hDAT T356M* under the background of the *Drosophila DAT* gene mutation induced hyperlocomotion activity in adult flies that was monitored by the activity assay (Trikinetics), reflecting a characteristic of ASD [94].

Linkage analyses, genome-wide association studies, and microarray analyses have identified some chromosomal loci carrying microdeletions or microduplications that are strongly associated with ASD. The recently identified microdeletions and microduplications of 16p11.2 have been observed in several familial studies and are associated with 0.5~1% of ASD cases [149]. The 16p11.2 microdeletion has been sub-mapped to affect 24~27 annotated genes, including *KIF22* and *MAPK3* [150,151]; however, the loss-of-function loci responsible for the increased risk of ASD remain unknown. Lee et al. took a unique approach using *Drosophila* larvae and identified candidate ASD-related genes [152,153]. The knockdown of *klp68D*, a candidate homologue of human *KIF22* induced ectopic innervations of synapse branches forming type III boutons in muscle 13, accompanied by the less frequent re-routing of other synapse branches at the NMJ [152]. Furthermore, mutations in *klp64D*, the gene product of which forms the Kinesin-2 complex with KLP68D, led to similar targeting errors in synapses at the NMJ [152]. Thus, Kinesin-2 proteins, including KLP68D and KLP64D, play a crucial role in synapse formation at the NMJ in *Drosophila*, suggesting KIF22 as a candidate ASD-related gene; however, further analyses are necessary to address this point. The mutation of *rolled*, a *Drosophila* homolog of human mitogen-activated protein kinase 3 (MAPK3), induced the ectopic innervation of synapse branches and the premature de-fasciculation of presynaptic axon bundles, suggesting the crucial role of MAPK3 in the regulation of the accurate targeting of presynaptic axons to proper postsynaptic targets that may be altered in ASD [153].

Large numbers of patients with ASD, with unaffected family members, have been shown to possess de novo CNVs [154,155,156,157]. Many of these CNVs appear to affect genes operating in the same biological pathways [158]. Computational and statistical analyses have proposed a contribution from the combinatorial effects of genetic variations. However, these hypotheses have not been validated in vivo. Grice et al. used *Drosophila* as an in vivo system to examine genetic interactions that may contribute to the neurological phenotypes of ASD, with a focus on the *neurexin IV* gene, the orthologue of the human ASD gene *CNTNAP2* [5]. An evaluation of bouton numbers at the larval NMJ and circadian defects in adult sleep and wake cycles (Trikinetics) revealed that mutations in subsets of genes in CNVs may synergistically interact to cause similar neuronal changes to the single candidate gene and changes in synapse sizes follow the direction of the human gene copy number change, supporting multiple-hit models of ASD [5]. These genetic approaches with *Drosophila* are useful for revealing the mechanisms by which synergistic effects resulting from large structural variations in the genome contribute to complex diseases such as ASD and ASD-related syndromes.

## 5. Perspectives

In the past decade, extensive advances have been achieved in the molecular diagnosis of ASD and many ASD-associated genes have been identified. To perform the functional characterization of these genes, identify novel biomarkers for ASD and ASD-related syndromes, and study the molecular pathology underlying ASD and ASD-related syndromes, several animal models including primates, mice, *Drosophila*, and nematodes have been used. Among these model organisms, we compared mouse (*M. musculus*) and *Drosophila* (*D. melanogaster*) as summarized in Table 1. Considering the simplicity of genome and availability of large numbers of mutants and RNAi lines, *Drosophila* is more suitable to carry out genome wide genetic screen compared to mouse. Although *Drosophila* has a similar origin of the CNS and neurobiological processes, including membrane excitability, neuronal signaling, and neurotransmitters, mouse contains more neuronal cells and is suitable for the analysis of complex behavior compared to *Drosophila*. In addition, *Drosophila* has no Schwann cell and therefore it is not suitable to study Schwann cell-related neural functions as discussed previously with Charcot–Marie–Tooth disease models in *Drosophila* [98]. Less ethical concern in making experiments is another advantage for the *Drosophila* model, although it may depend on situations in each country. *Drosophila* produces more offspring compared to mouse, providing experimental systems with high throughput and low cost. 

Since many patients of ASD and ASD-related syndromes show multiple genetic variants, inherited variants may lead to ASD and ASD-related syndromes through the synergistic effects of distinct deleterious variants involving a common biological pathway. Therefore, further studies are needed to investigate the roles of multiple gene interactions in ASD and ASD-related syndromes. In compared to rodents, *Drosophila* is especially useful for analyzing these complex gene networks, which are important for understanding the genetic causes of ASD and ASD-related syndromes. *Drosophila* will definitely show its power in identifying multiple gene interactions, thereby revealing distinct molecular etiologies underlying ASD and ASD-related syndromes. The combinatorial use of various assays with *Drosophila* models, including an NMJ analysis, circadian analysis, learning analysis, and informatics-targeted screening, is needed. The onset of ASD and ASD-related syndromes is affected by various environmental factors. It is important to note that missense variants for genes encoding the H3K9-specific methyltransferases GLP/EHMT1 and G9a/EHMT2 are ASD-associated genes [17,18]. *Drosophila* is useful for clarifying how environmental stress exerts effects on epigenetic regulators and the *Drosophila* model for ASD and ASD-related syndromes will become an invaluable tool for examining the environmental factors of ASD and ASD-related syndromes. In addition, pharmacological assays to develop a potential treatment for ASD and ASD-related syndromes will be performed with *Drosophila* models that must be followed by assays with various mouse models.

## Figures and Tables

**Table 1:** Comparison of mouse (*M. musculus*) and *Drosophila* (*D. melanogaster*) as model organism.

	Mouse	*Drosophila*
Genome size	2.8 Gbp	0.14 Gbp
Protein coding shared with human	90%	50%
Matching genes associated with human diseases	65%	75%
Neuronal cells/brain	1 × 10^5^	7 × 10^7^
Complex behavior	+++	+
Generation time	50 days	10 days
Genome wide genetic screen	+	+++
Production of offspring/female	10	100
Ethical restriction	+++	+

## References

[1] Sztainberg Y., Zoghbi H.Y. (2016). Lessons learned from studying syndromic autism spectrum disorders. Nat. Neurosci..

[2] Yuen R.K., Merico D., Cao H., Pellecchia G., Alipanahi B., Thiruvahindrapuram B., Tong X., Sun Y., Cao D., Zhang T. (2016). Genome-wide characteristics of de novo mutations in autism. Genom. Med..

[3] Tian Y., Zhang Z.C., Han J. (2017). *Drosophila* studies on autism spectrum disorders. Nuerosci. Bull..

[4] Gatto C.L., Broadie K. (2011). *Drosophila* modeling of heritable neurodevelopmental disorders. Curr. Opin. Neurobiol..

[5] Grice S.J., Liu J.-L., Webber C. (2015). Synergistic interactions between *Drosophila* orthologues of genes spanned by de novo human CNVs support multiple-hit models of autism. PLoS Genet..

[6] Darnell J.C., Van Driesche S.J., Zhang C., Hung K.Y., Mele A., Fraser C.E., Stone E.F., Chen C., Fak J.J., Chi S.W. (2011). FMRP stalls ribosomal translocation on mRNAs linked to synaptic function and autism. Cell.

[7] Dindot S.V., Antalffy B.A., Bhattacharjee M.B., Beaudet A.L. (2008). The Angelman syndrome ubiquitin ligase localizes to the synapse and nucleus, and maternal deficiency results in abnormal dendritic spine morphology. Hum. Mol. Genet..

[8] Finucane B.M., Lusk L., Arkilo D., Chamberlain S., Devinsky O., Dindot S., Jeste S.S., Lasalle J.M., Reiter L.T., Schanen N.C., Pagon R.A., Adam M.P., Ardinger H.H., Wallace S.E., Amemia A., Bean L.J.H., Bird T.D., Ledbetter N., Mefford H.C., Smith R.J.H. (2016). 15q Duplication Syndrome and Related Disorders.

[9] Sanders S.J., Murtha M.T., Gupta A.R., Murdoch J.D., Raubeson M.J., Willsey A.J., Ercan-Sencicek A.G., DiLullo N.M., Parikshak N.N., Stein J.L. (2012). De novo mutations revealed by whole-exome sequencing are strongly associated with autism. Nature.

[10] O’Roak B.J., Vives L., Fu W., Egertson J.D., Stanaway I.B., Phelps I.G., Carvill G., Kumar A., Lee C., Ankenman K. (2012). Multiple targeted sequencing identifies recurrently mutated genes in autism spectrum disorders. Science.

[11] Neale B.M., Kou Y., Liu L., Ma’ayan A., Samocha K.E., Sabo A., Lin C.F., Stevens C., Wang L.S., Makarov V. (2012). Patterns and rates of exonic de novo mutations in autism spectrum disorders. Nature.

[12] Iossifov I., Ronemus M., Levy D., Wang Z., Hakker I., Rosenbaum J., Yamrom B., Lee Y.H., Narzisi G., Leotta A. (2012). De novo gene disruptions in children on the autistic spectrum. Neuron.

[13] Iossifov I., O’Roak B.J., Sanders S.J., Ronemus M., Krumm N., Levy D., Stessman H.A., Witherspoon K.T., Vives L., Patterson K.E. (2014). The contribution of de novo coding mutations to autism spectrum disorder. Nature.

[14] De Rubeis S., He X., Goldberg A.P., Poultney C.S., Samocha K., Cicek A.E., Kou Y., Liu L., Fromer M., Walker S. (2014). Synaptic transcriptional and chromatin genes disrupted in autism. Nature.

[15] Gilissen C., Hehir-Kwa J.Y., Thung D.T., van de Vorst M., van Bon B.W., Willemsen M.H., Kwint M., Janssen I.M., Hoischen A., Schenck A. (2014). Genome sequencing identifies major causes of severe intellectual disability. Nature.

[16] Fromer M., Pocklington A.J., Kavanagh D.H., Williams H.J., Dwyer S., Gormley P., Georgieva L., Rees E., Palta P., Ruderfer D.M. (2011). De novo mutations in schizophrenia implicate synaptic networks. Nature.

[17] Balan S., Iwayama Y., Maekawa M., Toyota T., Ohnishi T., Toyoshima M., Shimamoto C., Esaki K., Yamada K., Iwata Y. (2014). Exon resequencing of H3K9 methyltransferase complex genes, EHMT1, EHMT2 and WIZ, in Japanese autism subjects. Mol. Autism.

[18] Koemans T.S., Kleefstra T., Chubak M.C., Stone M.H., Reijnders M.R.F., de Munnik S., Willemsen M.H., Fenckova M., Stumpel C.T.R.M., Bok L.A. (2017). Functional convergence of histone methyltransferase EHMT1 and KMT2C involved in intellectual disability and autism spectrum disorder. PLoS Genet..

[19] Kilpinen H., Ylisaukko-Oja T., Hennah W., Palo O.M., Varilo T., Vanhala R., Nieminen-von Wendt T., von Wendt L., Paunio T., Peltonen L. (2008). Association of DISC1 with autism and Asperger syndrome. Mol. Psychiatr..

[20] Fishwick K.J., Li R.A., Halley P., Deng P., Storey K.G. (2010). Initiation of neuronal differentiation requires PI3-kinase/TOR signalling in the vertebrate neural tube. Dev. Biol..

[21] Orlova K.A., Crino P.B. (2010). The tuberous sclerosis complex. Ann. N. Y. Acad. Sci..

[22] Han S., Witt R.M., Santos T.M., Polizzano C., Sabatini B.L., Ramesh V. (2008). Pam (Protein associated with Myc) functions as an E3 ubiquitin ligase and regulates TSC/mTOR signaling. Cell Signal..

[23] Betancur C., Buxbaum J.D. (2013). SHANK3 haploinsufficiency: A ‘‘common’’ but underdiagnosed highly penetrant monogenic cause of autism spectrum disorders. Mol. Autism.

[24] Kolevzon A., Cai G., Soorya L., Takahashi N., Grodberg D., Kajiwara Y., Willner J.P., Tryfon A., Buxbaum J.D. (2011). Analysis of a purported SHANK3 mutation in a boy with autism: Clinical impact of rare variant research in neurodevelopmental disabilities. Brain Res..

[25] Arons M.H., Thynne C.J., Grabrucker A.M., Li D., Schoen M., Cheyne J.E., Boeckers T.M., Montgomery J.M., Garner C.C. (2012). Autism-associated mutations in ProSAP2/Shank3 impair synaptic transmission and neurexin-neuroligin-mediated transsynaptic signaling. J. Neurosci..

[26] Castermans D., Wilquet V., Parthoens E., Huysmans C., Steyaert J., Swinnen L., Fryns J.P., Van de Ven W., Devriendt K. (2003). The *neurobeachin* gene is disrupted by a translocation in a patient with idiopathic autism. J. Med. Genet..

[27] Savelyeva L., Sagulenko E., Schmitt J.G., Schwab M. (2006). The *neurobeachin* gene spans the common fragile site *FRA13A*. Hum. Genet..

[28] Bi C., Wu J., Jiamg T., Liu Q., Cai W., Yu P., Cai T., Zhao M., Jiang Y.H., Sun Z.S. (2012). Mutations of ANK3 identified by exome sequencing are associated with autism susceptibility. Hum. Mut..

[29] Smith K.R., Kopeikina K.J., Fawcett-Patel J.M., Leaderbrand K., Gao R., Schurmann B., Myczek K., Radulovic J., Swanson G.T., Penzes P. (2014). Psychiatric risk factor ANK3/Ankyrin-G nanodomains regulate the structure and function of glutamatergic synapses. Neuron.

[30] Poot M. (2015). Cennecting the CNTNAP2 networks with neurodevelopmental disorders. Mol. Syndromol..

[31] Canali G., Goutebroze L. (2018). *CNTNAP2* heterozygous missense variants: Risk factors for autism spectrum disorder and/or other pathologies?. J. Exp. Neurosci..

[32] Sudfof T.C. (2017). Synaptic neurexin complexes: Molecular code for the logic of neural circuits. Cell.

[33] Jamain S., Quach H., Betancur C., Rastam M., Colineaux C., Gillberg I.C., Soderstrom H., Giros B., Leboyer M., Gillberg C. (2003). Mutations of the X-linked genes encoding neuroligins NLGN3 and NLGN4 are associated with autism. Nat. Genet..

[34] Chaste P., Leboyer M. (2012). Autism risk factor: Genes, environment, and gene-environment interactions. Dialogues Clin. Neurosci..

[35] Auerbach B.D., Osterweil E.K., Bear M.F. (2011). Mutations causing syndromic autism define an axis of synaptic pathophysiology. Nature.

[36] Nakamura K., Anitha A., Yamada K., Tsujii M., Iwayama Y., Hattori E., Toyota T., Suda S., Takei N., Iwata Y. (2008). Genetic and expression analyses reveal elevated expression of syntaxin 1A (STX1A) in high functioning autism. Int. J. Neuropsychopharmacol..

[37] Hamilton P.J., Campbell N.G., Sharma S., Erreger K., Herborg Hansen F., Saunders C., Belovich A.N., Sahai M.A., Cook E.H., NIH ARRA Autism Sequencing Consortium (2013). De novo mutation in the dopamine transporter gene associates dopamine dysfunction with autism spectrum disorder. Mol. Psychiatry.

[38] Tomioka M., Toda Y., Kurisu J., Kimura Y., Kengaku M., Ueda K. (2012). The effects of neurological disorder-related codon variations of ABCA13 on the function of the ABC protein. Biosci. Biotechnol. Biochem..

[39] Knight H.M., Pickard B.S., Maclean A., Malloy M.P., Soares D.C., McRae A.F., Condie A., White A., Hawkins W., McGhee K. (2009). A cytogenetic abnormality and rare coding variants identify ABCA13 as a candidate gene in schizophrenia, bipolar disorder, and depression. Am. J. Hum. Genet..

[40] Yoshida K., Go Y., Kushima I., Toyoda A., Fujiyama A., Imai H., Saito N., Iriki A., Ozaki N., Isoda M. (2016). Single-neuron and genetic correlates of autistic behavior in macaque. Sci. Adv..

[41] Jobski K., Hofer J., Hoffmann F., Bachmann C. (2017). Use of psychotropic drugs in patients with autism spectrum disorders: A systematic review. Acta Psychiatr. Scand..

[42] Lord C., Risi S., Lambrecht L., Cook E.H., Leventhal B.L., DiLavore P.C., Pickles A., Rutter M. (2000). The autism diagnostic observation schedule-generic: A standard measure of social and communication deficits associated with the spectrum of autism. J. Autism Dev. Disord..

[43] Volkmar F.R., Pauls D. (2003). Autism. Lancet.

[44] Okada R., Fujiwara H., Mizuki D., Araki R., Yabe T., Matsumoto K. (2015). Involvement of dopaminergic and cholinergic systems in social isolation-induced deficits in social affiliation and conditional fear memory in mice. Neuroscience.

[45] Ebihara K., Fujiwara H., Awale S., Dibwe D.F., Araki R., Yabe T., Matsumoto K. (2017). Decrease in endogenous brain allopregnanolone induces autism spectrum disorder (ASD)-like behavior in mice: A novel animal model of ASD. Behav. Brain Res..

[46] Fujiwara H., Han Y., Ebihara K., Awale S., Araki R., Yabe T., Matsumoto K. (2017). Daily administration of yokukansan and keishito prevents social isolation-induced behavioral abnormalities and down-regulation of phosphorylation of neuroplasticity-related signaling molecules in mice. BMC Complement. Altern. Med..

[47] Silverman J.L., Yang M., Lord C., Crawley J.N. (2010). Behavioural phenotyping assays for mousemodels of autism. Nat. Rev. Neurosci..

[48] Ouchi H., Ono K., Murakami Y., Matsumoto K. (2013). Social isolation induces deficit of latent learning performance in mice: A putative animal model of attention deficit/hyperactivity disorder. Behav. Brain Res..

[49] Riedel G., Spink A., Veltkamp R. (2014). Measuring Behavior. J. Neurosci. Methods.

[50] Mamiya T., Noda Y., Nishi M., Takeshima H., Nabeshima T. (1998). Enhancement of spatial attention in nociceptin/orphanin FQ receptor-knockout mice. Brain Res..

[51] Gepner B., Feron F. (2009). Autism: A world changing too fast for a mis-wired brain?. Neurosci. Biobehav. Rev..

[52] Roullet F.I., Lai J.K., Foster J.A. (2013). In utero exposure to valproic acid and autism-a current review of clinical and animal studies. Neurotoxicol. Teratol..

[53] Eissa N., Al-Houqani M., Sadeq A., Ojha S.K., Sasse A., Sadek B. (2018). Current enlightenment about etiology and pharmacological treatment of autism spectrum disorder. Front. Neurosci..

[54] Bolte S., Girdler S., Marschik P.B. (2019). The contribution of environmental exposure to the etiology of autism spectrum disorder. Cell. Mol. Life Sci..

[55] Christianson A.L., Chesler N., Kromberg J.G.R. (1994). Fetal valproate syndrome: Clinical and neurodevelopmental features in two sibling pairs. Dev. Med. Child Neurol..

[56] Strömland K., Nordin V., Miller M.T., Akerstrom B., Gillberg C. (1994). Autism in thalidomide embryopathy: A population study. Dev. Med. Child Neurol..

[57] Nanson J.L. (1992). Autism in fetal alcohol syndrome: A report of six cases. Alcohol. Clin. Exp. Res..

[58] Moore S.J., Turnpenny P., Quinn A., Glover S., Lloyd D.J., Montgomery T., Dean J.C. (2000). A clinical study of 57 children with fetal anticonvulsant syndromes. J. Med. Genet..

[59] Meador K.J., Baker G.A., Browning N., Cohen M.J., Clayton-Smith J., Kalayjian L.A., Kanner A., Liporace J.D., Pennell P.B., Privitera M. (2011). NEAD Study Group. Foetal antiepileptic drug exposure and verbal versus non-verbal abilities at three years of age. Brain.

[60] Shallcross R., Bromley R.L., Irwin B., Bonnett L.J., Morrow J., Baker G.A. (2011). Child development following in utero exposure: Levetiracetam vs sodium valproate. Neurology.

[61] Bambini-Junior V., Rodrigues L., Behr G.A., Moreira J.C., Riesgo R., Gottfried C. (2011). Animal model of autism induced by prenatal exposure to valproate: Behavioral changes and liver parameters. Brain Res..

[62] Massa V., Cabrera R.M., Menegola E., Giavini E., Finnell R.H. (2005). Valproic acid-induced skeletal malformations: Associated gene expression cascades. Pharm. Genom..

[63] Kogan M.D., Vladutiu C.J., Schieve L.A., Ghandour R.M., Blumberg S.J., Zablotsky B., Perrin J.M., Shattuck P., Kuhlthau K.A., Harwood R.L. (2018). The prevalence of parent-reported autism spectrum disorder among US children. Pediatrics.

[64] Kim K.C., Kim P., Go H.S., Choi C.S., Park J.H., Kim H.J., Jeon S.J., Dela Pena I.C., Han S.H., Cheong J.H. (2013). Male-specific alteration in excitatory post-synaptic development and social interaction in pre-natal valproic acid exposure model of autism spectrum disorder. J. Neurochem..

[65] Kataoka S., Takuma K., Hara Y., Maeda Y., Ago Y., Matsuda T. (2013). Autism-like behaviours with transient histone hyperacetylation in mice treated prenatally with valproic acid. Int. J. Neuropsychopharmacol..

[66] Schneider T., Roman A., Basta-Kaim A., Kubera M., Budziszewska B., Schneider K., Przewłocki R. (2008). Gender-specific behavioral and immunological alterations in an animal model of autism induced by prenatal exposure to valproic acid. Psychoneuroendocrinology.

[67] Jeon S.J., Gonzales E.L., Mabunga D.F.N., Valencia S.T., Kim D.G., Kim Y., Adil K.J.L., Shin D., Park D., Shin C.Y. (2018). Sex-specific Behavioral Features of Rodent Models of Autism Spectrum Disorder. Exp. Neurobiol..

[68] Kuo H.Y., Liu F.C. (2018). Molecular pathology and pharmacological treatment of autism spectrum disorder-like phenotypes using rodent models. Front. Cell Neurosci..

[69] Puia G., Mienville J.M., Matsumoto K., Takahata H., Watanabe H., Costa E., Guidotti A. (2003). On the putative physiological role of allopregnanolone on GABAA receptor function. Neuropharmacology.

[70] Coghlan S., Horder J., Inkster B., Mendez M.A., Murphy D.G., Nutt D.J. (2012). GABA system dysfunction in autism and related disorders: From synapse to symptoms. Neurosci. Biobehav. Rev..

[71] Cellot G., Cherubini E. (2014). GABAergic signaling as therapeutic target for autism spectrum disorders. Front. Pediatr..

[72] Guo Q.-Y., Ebihara K., Shimodaira T., Fujiwara H., Toume K., Dibwe D.F., Awale S., Araki R., Yabe T., Matsumoto K. (2019). Kami-shoyo-san improves ASD-like behaviors caused by decreasing allopregnanolone biosynthesis in an SKF mouse model of autism. PLoS ONE.

[73] Shinoda Y., Sadakata T., Furuichi T. (2013). Animal models of autism spectrum disorder (ASD): A synaptic-level approach to autistic-like behavior in mice. Exp. Anim..

[74] de la Torre-Ubieta L., Won H., Stein J.L., Geschwind D.H. (2016). Advancing the understanding of autism disease mechanisms through genetics. Nat. Med..

[75] Hubert S.W., Jiang Y.H. (2016). Monogenic mouse models of autism spectrum disorders: Common mechanisms and missing links. Neuroscience.

[76] Huber K.M., Gallagher S.M., Warren S.T., Bear M.F. (2002). Altered synaptic plasticity in a mouse model of fragile X mental retardation. Proc. Natl. Acad. Sci. USA.

[77] Dahlhaus R., El-Husseini A. (2010). Altered neuroligin expression is involved in social deficits in a mouse model of the fragile X syndrome. Behav. Brain Res..

[78] Spencer C.M., Alekseyenko O., Hamilton S.M., Thomas A.M., Serysheva E., Yuva-Paylor L.A., Paylor R. (2011). Modifying behavioral phenotypes in *Fmr1* KO mice: Genetic background difference reveal autistic-like responses. Autism Res..

[79] Chao H.T., Chen H., Samaco R.C., Xue M., Chahrour M., Yoo J., Neul J.L., Gong S., Lu H.C., Heintz N. (2010). Dysfunction in GABA signalling mediates autism-like stereotypies and Rett syndrome phenotypes. Nature.

[80] Samaco R.C., McGraw C.M., Ward C.S., Sun Y., Neul J.L., Zoghbi H.Y. (2012). Female Mecp2+/− mice display robust behavioral deficits on two different genetic backgrounds providing a framework for pre-clinical studies. Hum. Mol. Genet..

[81] Fukuda T., Itoh M., Ichikawa T., Washiyama K., Goto Y.J. (2005). Delayed maturation of neuronal architecture and synaptogenesis in cerebral cortex of Mecp2-deficient mice. Neuropathol. Exp. Neurol..

[82] Kline D.D., Ogier M., Kunze D.L., Katz D.M. (2010). Exogenous brain-derived neurotrophic factor rescues synaptic dysfunction in Mecp2-null mice. J. Neurosci..

[83] Jiang Y.H., Armstrong D., Albrecht U., Atkins C.M., Noebels J.L., Eichele G., Sweatt J.D., Beaudet A.L. (1998). Mutation of the Angelman ubiquitin ligase in mice causes increased cytoplasmic p53 and deficits of contextual learning and long-term potentiation. Neuron.

[84] Miao S., Chen R., Ye J., Tan G.H., Li S., Zhang J., Jiang Y.H., Xiang Z.Q. (2013). The Angelman syndrome protein Ube3a is required for polarized dendrite morphogenesis in pyramidal neurons. J. Neurosci..

[85] Greer P.L., Hanayama R., Bloodgood B.L., Mardinly A.R., Lipton D.M., Flavell S.W., Kim T.K., Griffith E.C., Waldon Z., Maehr R. (2010). The Angelman syndrome protein Ube3A regulates synapse development by ubiquitinating arc. Cell.

[86] Cao C., Rioult-Pedotti M.S., Migani P., Yu C.J., Tiwari R., Parang K., Spaller M.R., Goebel D.J., Marshall J. (2013). Impairment of TrkB-PSD-95 signaling in Angelman syndrome. PLoS Biol..

[87] Bey A.L., Jiang Y.H. (2014). Overview of mouse models of autism spectrum disorders. Curr. Protoc. Pharmacol..

[88] Crino P.B. (2013). Evolving neurobiology of tuberous sclerosis complex. Acta Neuropathol..

[89] Nuytens K., Gantois I., Stijnen P., Iscru E., Laeremans A., Serneels L., Van Eylen L., Liebhaber S.A., Devriendt K., Balschun D. (2013). Haploinsufficiency of the autism candidate gene Neurobeachin induces autism-like behaviors and affects cellular and molecular processes of synaptic plasticity in mice. Neurobiol. Dis..

[90] Niesmann K., Breuer D., Brockhaus J., Born G., Wolff I., Reissner C., Kilimann M.W., Rohlmann A., Missler M. (2011). Dendritic spine formation and synaptic function require neurobeachin. Nat. Commun..

[91] Nair R., Lauks J., Jung S., Cooke N.E., de Wit H., Brose N., Kiliman M.W., Verhage M., Rhee J. (2013). Neurobeachin regulates neurotransmitter receptor trafficking to synapse. J. Cell Biol..

[92] Volders K., Nuytens K., Creemers J.W. (2011). The autism candidate gene Neurobeachin encodes a scaffolding protein implicated in membrane trafficking and signaling. Curr. Mol. Med..

[93] Simon A.F., Chou M.T., Salazar E.D., Nicholson T., Saini N., Metchev S., Krantz D.E. (2012). A simple assay to study social behavior in *Drosophila*: Measurement of social space within a group. Genes Brain Behav..

[94] Hamilton P.J., Campbell N.G., Sharma S., Erreger K., Hansen F.H., Saunders C., Belovich A.N., Sahai M.A., Cook E.H., Gether U. (2013). *Drosophila melanogaster*: A novel animal model for the behavioral characterization of autism-associated mutations in the dopamine transporter gene. Mol. Psychiatry.

[95] Ueoka I., Kawashima H., Konishi A., Aoki M., Tanaka R., Yoshida H., Maeda T., Ozaki M., Yamaguchi M. (2018). Novel *Drosophila* model for psychiatric disorders including autism spectrum disorder by targeting of ATP-binding cassette protein A. Exp. Neurol..

[96] Gerber B., Biernacki R., Thum J. (2013). Oor-taste learning assays in *Drosophila* larvae. Cold Spring Harb. Protoc..

[97] Jantrapirom S., Lo Piccolo L., Yoshida H., Yamaguchi M. (2018). A new *Drosophila* model of *Ubiquilin* knockdown shows the effect of impaired proteostasis on locomotive and learning abilities. Exp. Cell. Res..

[98] Yamaguchi M., Takashima H. (2018). *Drosophila* Charcot-Marie-Tooth disease models. Adv. Exp. Med. Biol..

[99] Brent J.R., Werner K.M., McCabe B.D. (2009). *Drosophila* larval NMJ dissection. J. Vis. Exp..

[100] Potikanond S., Nimlamool W., Noordermeer J., Fradkin L.G. (2018). Muscular Dystrophy Model. Adv. Exp. Med. Biol..

[101] Wan L., Dockendorff T.C., Jongens T.A., Dreyfuss G. (2000). Characterization of dFMR1, a *Drosophila melanogaster* homolog of the fragile X mental retardation protein. Mol. Cell Biol..

[102] Zhang Y.Q., Bailey A.M., Matthies H.J., Renden R.B., Smith M.A., Speese S.D., Rubin G.M., Broadie K. (2001). *Drosophila* fragile X-related gene regulates the MAP1B homolog Futsch to control synaptic structure and function. Cell.

[103] Morales J., Hiesinger P.R., Schroeder A.J., Kume K., Verstreken P., Jackson F.R., Nelson D.L., Hassan B.A. (2002). *Drosophila* fragile X protein, DFXR, regulates neuronal morphology and function in the brain. Neuron.

[104] Pan L., Zhang Y.Q., Woodruff E., Broadie K. (2004). The *Drosophila* fragile X gene negatively regulates neuronal elaboration and synaptic differentiation. Curr. Biol..

[105] Coffee R.L., Tessier C.R., Woodruff E.A., Broadie K. (2010). Fragile X mental retardation protein has a unique, evolutionarily conserved neuronal function not shared with FXR1P or FXR2P. Dis. Model. Mech..

[106] Gatto C.L., Broadie K. (2008). Temporal requirements of the fragile X mental retardation protein in the regulation of synaptic structure. Development.

[107] Lee A., Li W., Xu K., Bogert B.A., Su K., Gao F.B. (2003). Control of dendritic development by the *Drosophila* fragile X-related gene involves the small GTPase Rac1. Development.

[108] Xu X.L., Li Y., Wang F., Gao F.B. (2008). The steady-state level of the nervous-system-specific microRNA-124a is regulated by dFMR1 in *Drosophila*. J. Neurosci..

[109] Dockendorff T.C., Su H.S., McBride S.M., Yang Z., Choi C.H., Siwicki K.K., Sehgal A., Jongens T.A. (2002). *Drosophila* lacking dfmr1 activity show defects in circadian output and fail to maintain courtship interest. Neuron.

[110] Tessier C.R., Broadie K. (2008). *Drosophila* fragile X mental retardation protein developmentally regulates activity-dependent axon pruning. Development.

[111] Michel C.I., Kraft R., Restifo L.L. (2004). Defective neuronal development in the mushroom bodies of *Drosophila* fragile X mental retardation 1 mutants. J. Neurosci..

[112] Kashima R., Roy S., Ascano M., Martinez-Cerdeno V., Ariza-Torres J., Kim S., Louie J., Lu Y., Leyton P., Bloch K.D. (2016). Augmented noncanonical BMP type II receptor signaling mediates the synaptic abnormality of fragile X syndrome. Sci. Signal..

[113] Cvetkovska V., Hibbert A.D., Emran F., Chen B.E. (2013). Overexpression of Down syndrome cell adhesion molecule impairs precise synaptic targeting. Nat. Neurosci..

[114] Sudhakaran I.P., Hillebrand J., Dervan A., Das S., Holohan E.E., Hülsmeier J., Sarov M., Parker R., VijayRaghavan K., Ramaswami M. (2014). FMRP and Ataxin-2 function together in long-term olfactory habituation and neuronal translational control. Proc. Natl. Acad. Sci. USA.

[115] Bhogal B., Jepson J.E., Savva Y.A., Pepper A.S., Reenan R.A., Jongens T.A. (2011). Modulation of dADAR-dependent RNA editing by the *Drosophila* fragile X mental retardation protein. Nat. Neurosci..

[116] Madrigal I., Rodríguez-Revenga L., Xunclà M., Milà M. (2012). 15q11.2 microdeletion and FMR1 premutation in a family with intellectual disabilities and autism. Gene.

[117] Waltes R., Duketis E., Knapp M., Anney R.J.L., Huguet G., Schlitt S., Jarczok T.A., Sachse M., Kämpfer L.M., Kleinböck T. (2014). Common variants in genes of the postsynaptic FMRP signaling pathway are risk factors for autism spectrum disorders. Hum. Genet..

[118] Huang Y. (2015). Up-regulated cytoplasmic FMRP-interacting protein 1 in intractable temporal lobe epilepsy patients and a rat model. Int. J. Neurosci..

[119] Wang J., Tao Y., Song F., Sun Y., Ott J., Saffen D. (2015). Common regulatory variants of CYFIP1 contribute to susceptibility for Autism Spectrum Disorder (ASD) and classical autism. Ann. Hum. Genet..

[120] Abekhoukh S., Sahin H.B., Grossi M., Zongaro S., Maurin T., Madrigal I., Kazue-Sugioka D., Raas-Rothschild A., Doulazmi M., Carrera P. (2017). New insights into the regulatory function of CYFIP1 in the context of WAVE- and FMRP-containing complexes. Dis. Model. Mech..

[121] Drozd M., Bardoni B., Capovilla M. (2018). Modeling fragile X syndrome in *Drosophila*. Front. Mol. Neurosci..

[122] Johnson K.G., Tenney A.P., Ghose A., Duckworth A.M., Higashi M.E., Parfitt K., Marcu O., Heslip T.R., Marsh J.L., Schwarz T.L. (2006). The HSPGs Syndecan and Dallylike bind the receptor phosphatase LAR and exert distinct effects on synaptic development. Neuron.

[123] Friedman S.H., Dani N., Rushton E., Broadie K. (2013). Fragile X mental retardation protein regulates trans-synaptic signaling in *Drosophila*. Dis. Model Mech..

[124] Choi C.H., McBride S.M.J., Schoenfeld B.P., Liebelt D.A., Ferreiro D., Ferrick N.J., Hinchey P., Kollaros M., Rudominer R.L., Terlizzi A.M. (2010). Age-dependent cognitive impairment in a *Drosophila* fragile X model and its pharmacological rescue. Biogerontology.

[125] Kashima R., Redmond P.L., Ghatpande P., Roy S., Kornberg T.B., Hanke T., Knapp S., Lagna G., Hata A. (2017). Hyperactive locomotion in a *Drosophila* model is a functional readout for the synaptic abnormalities underlying fragile X syndrome. Sci. Signal..

[126] Monyak R.E., Emerson D., Schoenfeld B.P., Zheng X., Chambers D.B., Rosenfelt C., Langer S., Hinchey P., Choi C.H., McDonald T.V. (2016). Insulin signaling misregulation underlies circadian and cognitive deficits in a *Drosophila* fragile X model. Mol. Psychiatry.

[127] Reiter L.T., Seagroves T.N., Bowers M., Bier E. (2006). Expression of the Rho-GEF Pbl/ECT2 is regulated by the UBE3A E3 ubiquitin ligase. Hum. Mol. Genet..

[128] Wu Y., Bolduc F.V., Bell K., Tully T., Fang Y., Sehgal A., Fischer J.A. (2008). A *Drosophila* model for Angelman syndrome. Proc. Natl. Acad. Sci. USA.

[129] Lu Y., Wang F., Li Y., Ferris J., Lee J.A., Gao F.B. (2009). The *Drosophila* homologue of the Angelman syndrome ubiquitin ligase regulates the formation of terminal dendritic branches. Hum. Mol. Genet..

[130] Chakraborty M., Paul B.K., Nayak T., Das A., Jana N.R., Bhutani S. (2015). The E3 ligase ube3a is required for learning in *Drosophila melanogaster*. Biochem. Biophys. Res. Commun..

[131] Hope K.A., LeDoux M.S., Reiter L.T. (2017). Glial overexpression of Dube3a causes seizures and synaptic impairments in *Drosophila* concomitant with down regulation of the Na^+^/K^+^ pump ATPα. Neurobiol. Dis..

[132] Valdez C., Scroggs R., Chassen R., Reiter L.T. (2015). Variation in Dube3a expression affects neurotransmission at the *Drosophila* neuromuscular junction. Biol. Open.

[133] Ferdousy F., Bodeen W., Summers K., Doherty O., Wright O., Elsisi N., Hilliard G., O’Donnell J.M., Reiter L.T. (2011). *Drosophila* Ube3a regulates monoamine synthesis by increasing GTP cyclohydrolase I activity via a non-ubiquitin ligase mechanism. Neurobiol. Dis..

[134] Bucan M., Abrahams B.S., Wang K., Glessner J.T., Herman E.I., Sonnenblick L.I., Alvarez Retuerto A.I., Imielinski M., Hadley D., Bradfield J.P. (2009). Genome-wide analyses of exonic copy number variants in a family-based study point to novel autism susceptibility genes. PLoS Genet..

[135] Glessner J.T., Wang K., Cai G., Korvatska O., Kim C.E., Wood S., Zhang H., Estes A., Brune C.W., Bradfield J.P. (2009). Autism genome-wide copy number variation reveals ubiquitin and neuronal genes. Nature.

[136] Lee S.Y., Ramirez J., Franco M., Lectez B., Gonzalez M., Barrio R., Mayor U. (2014). Ube3a, the E3 ubiquitin ligase causing Angelman syndrome and linked to autism, regulates protein homeostasis through the proteasomal shuttle Rpn10. Cell. Mol. Life. Sci..

[137] Tuand K., Stijnen P., Volders K., Declercq J., Nuytens K., Meulemans S., Creemers J. (2016). Nuclear localization of the autism candidate gene *neurobeachin* and functional interaction with the NOTCH1 intracellular domain indicate a role in regulating transcription. PLoS ONE.

[138] Volders K., Scholz S., Slabbaert J.R., Nagel A.C., Verstreken P., Creemers J.W.M., Callaerts P., Schwarzel M. (2012). *Drosophila rugose* is a functional homolog of mammalian *neurobeachin* and affects synaptic architecture, brain morphology, and associative learning. J. Neurosci..

[139] Wise A., Tenezaca L., Fernandez R.W., Schatoff E., Flores J., Ueda A., Zhong X., Wu C.-F., Simon A.F., Venkatesh T. (2015). *Drosophila* mutants of the autism candidate gene *neurobeachin* (*rugose*) exhibit neuro-developmental disorders, aberrant synaptic properties, altered locomotion, impaired adult social behavior and activity patterns. J. Neurogenet..

[140] Shamloula H.K., Mbogho M.P., Pimentel A.C., Chrzanowska-Lightowlers Z.M.A., Hyatt V., Okano H., Venkatesh T.R. (2002). *Rugose* (*rg*), a *Drosophila* A kinase anchor protein, is required for retinal pattern formation and interacts genetically with multiple signaling pathways. Genetics.

[141] Wech I., Nagel A.C. (2005). Mutations in *rugose* promote cell type-specific apoptosis in the *Drosophila* eye. Cell Death Differ..

[142] Iritani S., Torii Y., Habuchi C., Sekiguchi H., Fujishiro H., Yoshida M., Go Y., Iriki A., Isoda M., Ozaki N. (2018). The neuropathological investigation of the brain in a monkey model of autism spectrum disorder with ABCA13 deletion. Int. J. Dev. Neurosci..

[143] Piehler A.P., Özcurumez M., Kaminski W.E. (2012). A-subclass ATP-binding cassette proteins in brain lipid homeostasis and neurodegeneration. Front. Psychiatry.

[144] Seeman P., Niznik H.B. (1990). Dopamine receptors and transporters in Parkinson’s disease and schizophrenia. FASEB J..

[145] Volkow N.D., Wang G.J., Newcorn J., Telang F., Solanto M.V., Fowler J.S., Logan J., Ma Y., Schulz K., Pradhan K. (2007). Depressed dopamine activity in caudate and preliminary evidence of limbic involvement in adults with attention-deficit/hyperactivity disorder. Arch. Gen. Psychiatry.

[146] Gadow K.D., Roohi J., DeVincent C.J., Hatchwell E. (2008). Association of ADHD, tics, and anxiety with dopamine transporter (DAT1) genotype in autism spectrum disorder. J. Child Psychol. Psychiatry.

[147] Cousins D.A., Butts K., Young A.H. (2009). The role of dopamine in bipolar disorder. Bipolar Disord..

[148] Nakamura K., Sekine Y., Ouchi Y., Tsujii M., Yoshikawa E., Futatsubashi M., Tsuchiya K.J., Sugihara G., Iwata Y., Suzuki K. (2010). Brain serotonin and dopamine transporter bindings in adults with high-functioning autism. Arch. Gen. Psychiatry.

[149] Weiss L.A., Shen Y., Korn J.M., Arking D.E., Miller D.T., Fossdal R., Saemundsen E., Stefansson H., Ferreira M.A., Green T. (2008). Association between microdeletion and microduplication at 16p11.2 and autism. N. Engl. J. Med..

[150] Kumar R.A., KaraMohamed S., Sudi J., Conrad D.F., Brune C., Badner J.A., Gilliam T.C., Nowak N.J., Cook E.H., Dobyns W.B. (2008). Recurrent 16p11.2 microdeletions in autism. Hum. Mol. Genet..

[151] Horev G., Ellegood J., Lerch J.P., Son Y.E., Muthuswamy L., Vogel H., Krieger A.M., Buja A., Henkelman R.M., Wigler M. (2011). Dosage-dependent phenotypes in models of 16p11.2 lesions found in autism. Proc. Natl. Acad. Sci. USA.

[152] Park S.M., Littleton J.T., Park H.R., Lee J.H. (2016). *Drosophila* homolog of human KIF22 at the autism-linked 16p11.2 loci influences synaptic connectivity at larval neuromuscular junctions. Exp. Neurobiol..

[153] Park S.M., Park H.R., Lee J.H. (2017). MAPK3 at the autism-linked human 15p11.2 locus influences precise synaptic target selection at *Drosophila* larval neuromuscular junctions. Mol. Cells.

[154] Kusenda M., Sebat J. (2008). The role of rare structural variants in the genetics of autism spectrum disorders. Cytogenet. Genome Res..

[155] Pinto D., Pagnamenta A.T., Klei L., Anney R., Merico D., Regan R., Conroy J., Magalhaes T.R., Correia C., Abrahams B.S. (2010). Functional impact of global rare copy number variation in autism spectrum disorders. Nature.

[156] Moreno-De-Luca D., Sanders S.J., Willsey A.J., Mulle J.G., Lowe J.K., Geschwind D.H., State M.W., Martin C.L., Ledbetter D.H. (2013). Using large clinical data sets to infer pathogenicity for rare copy number variants in autism cohorts. Mol. Psychiatry.

[157] Sandin S., Lichtenstein P., Kuja-Halkola R., Larsson H., Hultman C.M., Reichenberg A. (2014). The familial risk of autism. JAMA.

[158] Noh H.J., Ponting C.P., Boulding H.C., Meader S., Betancur C., Buxbaum J.D., Pinto D., Marshall C.R., Lionel A.C., Scherer S.W. (2013). Network topologies and convergent aetiologies arising from deletions and duplications observed in individuals with autism. PLoS Genet..

