# Correction: Ueoka, I., et al. Autism Spectrum Disorder-Related Syndromes: Modeling with *Drosophila* and Rodents. *Int. J. Mol. Sci.* 2019, *20*, 4071

**DOI:** 10.3390/ijms21218093

**Published:** 2020-10-30

**Authors:** Ibuki Ueoka, Hang Thi Nguyet Pham, Kinzo Matsumoto, Masamitsu Yamaguchi

**Affiliations:** 1 Department of Applied Biology, Kyoto Institute of Technology, Matsugasaki, Sakyo-ku, Kyoto 603-8585, Japan; merry.christmas.ibu@gmail.com; 2 Department of Pharmacology and Biochemistry, National Institute of Medicinal Materials, Hanoi 110100, Vietnam; pnhang2004@yahoo.com; 3 Division of Medicinal Pharmacology, Institute of Natural Medicine, University of Toyama, Toyama 930-0194, Japan; kinzo.matsumoto@gmail.com

The author wishes to make the following correction to this paper [1]. Due to mistakes in numerical values (“Protein coding genes shared with human”, “% of human disease genes conserved”, “Neuronal cells/brain”, and “Production of offspring/female”) in Table 1, it should be replaced with the following table:

These changes have no material impact on the conclusions of our paper. The authors apologize for any inconvenience caused to the readers by these changes.

## Figures and Tables

**Table 1:** Comparison of mouse (*M. musculus*) and *Drosophila* (*D. melanogaster*) as model organism.

	Mouse	*Drosophila*
Genome size	2.8 Gbp	0.14 Gbp
Protein coding genes shared with human	88%	58%
% of human disease genes conserved	99%	75%
Neuronal cells/brain	7 × 10^7^	1.35 × 10^5^
Complex behavior	+++	+
Generation time	50 days	10 days
Genome-wide genetic screen	+	+++
Production of offspring/female	10/litter	100 eggs/day
Ethical restriction	+++	+
